# Effect of Diacerein on Metabolic Control and Inflammatory Markers in Patients with Type 2 Diabetes Using Antidiabetic Agents: A Randomized Controlled Trial

**DOI:** 10.1155/2018/4246521

**Published:** 2018-04-02

**Authors:** Glaucia S. Tres, Sandra C. Fuchs, Fabiana Piovesan, Patricia Koehler-Santos, Fernanda dos S. Pereira, Suzi Camey, Hugo K. Lisboa, Leila B. Moreira

**Affiliations:** ^1^Postgraduate Program in Cardiology, School of Medicine, Universidade Federal do Rio Grande do Sul (UFRGS), R. Ramiro Barcelos 2600, 90035-003 Porto Alegre, RS, Brazil; ^2^Hospital São Vicente de Paulo, School of Medicine, Universidade de Passo Fundo (UPF), R. Teixeira Soares 808, 99010-080 Passo Fundo, RS, Brazil; ^3^Unidade de Análises Moleculares e de Proteínas (UAMP), Hospital de Clinicas de Porto Alegre, Universidade Federal do Rio Grande do Sul (UFRGS), R. Ramiro Barcelos 2350, 90035-903 Porto Alegre, RS, Brazil; ^4^Department of Statistics, Mathematics Institute, Universidade Federal do Rio Grande do Sul, R. Ramiro Barcelos 2350, 90035-903 Porto Alegre, RS, Brazil; ^5^Biostatistics Unit, GPPG, Hospital de Clinicas de Porto Alegre, Universidade Federal do Rio Grande do Sul (UFRGS), R. Ramiro Barcelos 2350, 90035-903 Porto Alegre, RS, Brazil

## Abstract

**Introduction:**

Studies have shown that T2DM is an inflammatory disease. Thus, the present study was aimed at evaluating whether diacerein could improve the metabolic and inflammatory profile among patients with T2DM under long-term treatment with glucose-lowering agents.

**Methods:**

This is a double-blind, parallel, placebo-controlled trial with 72 participants randomly assigned to diacerein 50 mg or placebo for 12 weeks. The primary endpoint was the between-group difference in change in HbA1c. Secondary endpoints included the proportion of patients achieving metabolic control [HbA1c ≤ 7.0% (53 mmol/mol)] and change in inflammatory mediators.

**Results:**

Participants in the diacerein group had greater reductions in mean HbA1c level in comparison to placebo (−0.98; 95% CI: −2.02 to 0.05, *P* = 0.06), independently of confounding factors. The difference in HbA1c level was −1.3 (95% CI: −2.3 to −0.4) in favor of diacerein (*P* = 0.007) in those with <14 years of diabetes duration versus 0.05 (−0.7 to 0.8; *P* = 0.9) in those with longer duration. The diacerein group had a 50% increase in the number of participants at the lowest TNF-*α* level (≤1.46 pg/mL).

**Conclusions:**

In patients with long-established T2DM under long-term treatment with glucose-lowering agents, diacerein improves metabolic control as measured by HbA1c level and has a favorable impact on inflammatory profile.

**Clinical Trial Registry:**

This trial is registered with Brazilian Clinical Trials Registry (ReBEC) number RBR-29j956.

## 1. Introduction

Type 2 diabetes mellitus (T2DM) and obesity-related insulin resistance (IR) have been involved in a state of low-grade chronic inflammation and activation of the immune system [1]. The size of adipocytes might play a role in IR and lipolytic activity, since larger ones release less adiponectin and more inflammatory cytokines [2]. The immune changes that occur in diabetes—changes in the number and activation state of leukocyte populations, increased apoptosis and tissue fibrosis, abnormal cytokine production, elevated levels of acute-phase proteins, and other mediators—have been shown to activate inflammatory pathways [3–5]. Inflammation is related to insulin resistance, which increases pancreatic islet cell mass and insulin secretory activity until insulin deficiency develops [4, 6].

Several mechanisms contribute to beta-cell dysfunction and insulin resistance in T2DM. These mechanisms are strongly interlinked and contribute to tissue inflammation. The main dysfunctions affecting beta cells are glucotoxicity, lipotoxicity, oxidative stress, and amyloid deposition. All lead to activation of the immune system in adipose tissues, liver, pancreatic islets, the vasculature, and leukocytes. Immune adaptations alter levels of cytokines and chemokines, activate leukocyte populations, and increase apoptosis and tissue fibrosis [4].

The inflammatory pathway of T2DM has led to the investigation of drugs that act at different points of the inflammatory orbit. Anti-inflammatory treatment appears to improve insulin sensitivity and glycemic control in insulin-resistant patients with T2DM or inflammatory conditions [7]. In addition, glucose-lowering agents have been associated with anti-inflammatory effects, although it is not known whether this results from better glucose control or their intrinsic pharmacological effect [8]. Recent trials [9–11] of the anti-inflammatory agent diacerein conducted in patients with T2DM have shown improvement on metabolic control in both drug-naïve patients [9] and those under glucose-lowering therapy [10, 11]. Furthermore, diacerein seems to slow deterioration of metabolic control in patients with T2DM and chronic kidney disease [12].

However, most of these studies enrolled T2DM patients within a limited range of age and diabetes duration. In addition, there are few studies assessing the impact of diacerein on the metabolic and anti-inflammatory profile among patients with established disease undergoing long-term treatment, including with insulin. Within this context, the present study was aimed at evaluating whether diacerein could improve the metabolic and inflammatory profile of patients with T2DM under long-term treatment with glucose-lowering agents.

## 2. Materials and Methods

Patients with T2DM were recruited from the outpatient diabetes clinic of a university hospital in Passo Fundo, Southern Brazil. This randomized, double-blind, parallel, placebo-controlled trial enrolled participants aged 34 to 79 years, who had glycated hemoglobin (HbA1c) ≥ 7.4% (57.4 mmol/mol) and <11.0% (97 mmol/mol) and did not have kidney disease. Exclusion criteria included pregnancy, chronic inflammatory diseases such as rheumatoid arthritis and osteoarthritis, use of anti-inflammatory agents for more than five days in the last three months, previous diagnosis of pancreatitis, hypersensitivity to rhein and anthraquinone derivatives, liver disease (transaminases > 2.5 times the upper limit of normal), severe gastrointestinal disease/persistent diarrhea, signs of intercurrent infection, participation in another randomized controlled trial in the last 30 days, or inability to follow the protocol.

The protocol was registered into the Plataforma Brasil clinical trial registry as a precondition for Ethics Committee submission. It was approved by the Ethics Committee of Hospital de Clínicas de Porto Alegre (GPPG number 120482, on December 17, 2012), which is accredited by the US Office of Human Research Protections as an Institutional Review Board. The trial was subsequently registered in the Brazilian Clinical Trials Registry platform (available at http://www.ensaiosclinicos.gov.br/rg/RBR-29j956). Written informed consent was obtained from all participants, according to the principles expressed in the Declaration of Helsinki. This trial was designed, implemented, and described following the provisions of the CONSORT statement.

Participants were randomly assigned in a 1 : 1 ratio to receive either diacerein 50 mg or placebo, twice daily for 12 weeks. The randomization sequence was generated by a web-based randomization software (Random Allocation Software) with permuted-block allocation, with block sizes of four and six. This was done before the start of the trial by an epidemiologist not involved with clinical procedures. Diacerein and placebo capsules, identical in appearance, were stored in bottles labeled using an alphanumeric code. The bottles were stored out of the clinical site, with access controlled by a research assistant. After confirmation of eligibility, each participant received a bottle, ensuring allocation concealment and blinding of intervention to participants, care providers, and those assessing outcomes. Diacerein and placebo were manufactured by a pharmaceutical company (TRB Pharma, Campinas, SP, Brazil). Participants were asked to return for follow-up visits on days 7, 30, 60, and 90 after randomization to verify adherence to treatment and monitor adverse events. Patients in both groups were instructed to maintain their prescribed glucose-lowering agents and dietary recommendations. Adherence to treatment was self-reported and measured by counting the number of pills that were returned at each visit. The trial ended on day 90, when baseline measures were repeated.

The primary endpoint was the difference in change in HbA1c (end of trial minus baseline level), between the placebo and diacerein groups. Secondary endpoints included the proportion of patients achieving metabolic control [HbA1c ≤ 7.0% (53 mmol/mol)] and the difference between the two treatment arms in plasma levels of fasting glucose (mg/dL), inflammatory mediators [IL-1*β*, tumor necrosis factor- (TNF-) *α*, IL-6, and IL-10], and lipids [high-density lipoprotein cholesterol (HDLc), low-density lipoprotein cholesterol (LDLc), total cholesterol (TC), and triglycerides (TGs)], homeostasis model assessment of insulin resistance (HOMA1-IR), and rate of adverse events. Blood samples were collected after a 12-hour fast by venipuncture from all participants at baseline and at the end of the study. The samples were centrifuged and aliquoted for later analysis. Interleukin levels were measured using a customized Luminex® Human Ultrasensitive magnetic bead panel. Fasting glucose was measured by an enzymatic and colorimetric method and HbA1c by immunoturbidimetric assay.

The assessment included anthropometric measures, 24-hour ambulatory blood pressure monitoring (ABPM), use of statins, and blood pressure-lowering agents. Changes in the dose of glucose-lowering agents were collected in order to identify cointerventions that could affect the efficacy of the study intervention. Hypertension was defined as office systolic blood pressure (SBP) ≥ 140 mmHg or diastolic blood pressure (DBP) ≥ 90 mmHg or current treatment with blood pressure-lowering agents.

### 2.1. Sample Size Calculation and Statistical Analysis

A sample size of 31 participants in each group was estimated to detect a difference of 1.0% (SD ± 1.5) in HbA1c between diacerein and placebo arms, assuming a two-tailed test with significance of 0.05 and power of 80%. To account for possible losses to follow-up during the trial, 36 participants were included in each group. Outcomes were analyzed following the intention-to-treat approach.

Characteristics of the sample were compared between the two groups using Student's *t*-test for continuous variables or Pearson's chi-square test for categorical variables. A general linear model for baseline and end-of-trial measures was applied to analyze the effect of diacerein, and the change from baseline was estimated using analysis of covariance (ANCOVA) with control of baseline values for age, gender, diabetes duration, and change in dosage of glucose-lowering agents during the trial. *P* values < 0.05 were considered statistically significant. Because of the wide range of diabetes duration, analysis of HbA1c was stratified by diabetes duration (1–13 and 14–30 years) to explore the effect of diacerein in long-established diabetes mellitus. The levels of HbA1c were adjusted for gender and age. Considering the nonnormal distribution of inflammatory markers, they were categorized at the 25th or 75th percentile for baseline and end-of-trial values. The baseline 25th percentile was defined as the cutoff point for IL-6, IL-8, IL-10, leptin, selectin, and adiponectin, as well as the 75th percentile for IL-1*β*. For TNF-*α*, the 50th percentile (or median) baseline value was defined as the cutoff point. Poisson regression with a robust estimator was performed to verify the effect of diacerein on inflammatory markers, using the values at the end of the trial as outcomes and adjusting for baseline values. Relative risk and 95% CIs were used to express the association. All analyses were performed using SPSS for Windows (version 17.0; SPSS Inc., Chicago, IL, USA).

## 3. Results

**Figure 1:**
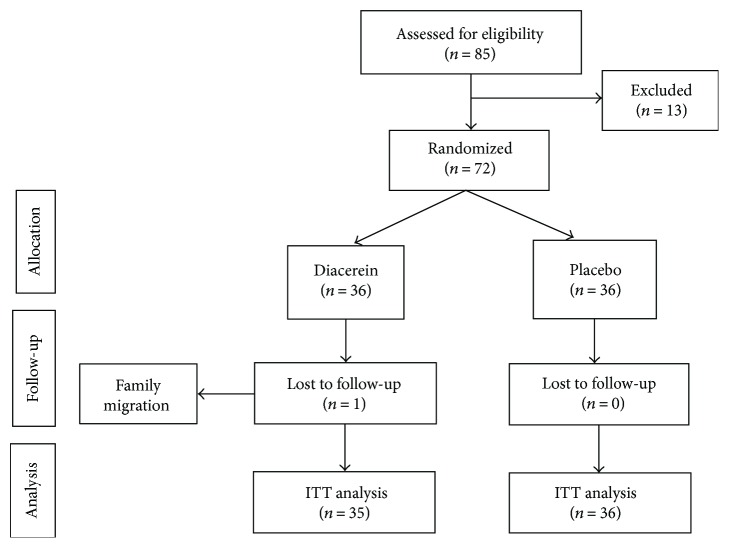
Flow diagram of participant selection, randomization, and follow-up.

Among 85 patients with T2DM assessed for eligibility, 72 were enrolled in the trial (36 in each group) and 71 completed the study. Participants attended all scheduled visits, but one participant in the diacerein group moved to another city and did not complete the baseline assessment (Figure 1). The trial was conducted from September 2013 to March 2014.

The baseline characteristics of the participants are shown in Table 1. The mean (SD) age was 62 (8) years in the diacerein group versus 59 (11) years in the placebo group. In both groups, participants, on average, completed six years of formal education, were obese, and had long-established diabetes, hypertension, and well-controlled blood pressure on 24-hour ABPM. Participants in the diacerein group were, on average, older, women, and with higher HbA1c levels, but used metformin less often than those who were taking placebo. About half of all participants were using insulin. Self-reported adherence to the study drug was high in both groups (diacerein, 93.7% ± 14.3; placebo, 92.7% ± 14.8), as well as pill counting, with 88.6% of diacerein-treated patients versus 86% of placebo-treated patients reporting 90% or more intake of the capsules. Although patients were advised to continue their glucose-lowering treatment as at baseline, one patient in the diacerein group and four in the placebo group had treatment modifications.

**Figure 2:**
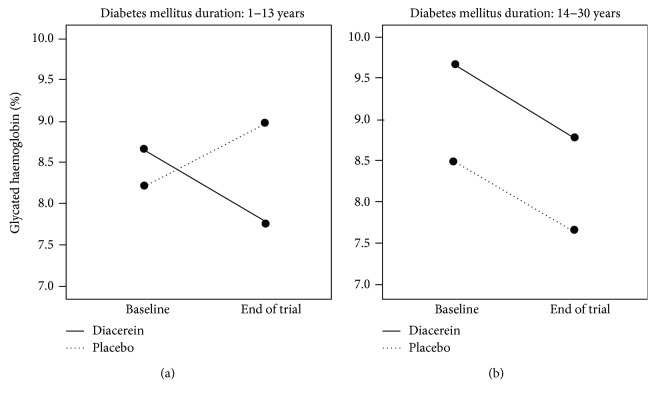
End-of-trial changes in HbA1c (%) in the diacerein and placebo groups, stratified by duration of diabetes < 14 (a) or ≥14 years (b), independently of age and gender.

Table 2 shows that participants in the diacerein group had greater reduction in mean HbA1c level compared to the placebo arm, independently of age, gender, duration of diabetes, and addition of a new antidiabetic drug. The adjusted difference in mean change (−0.98; 95% CI: −2.02 to 0.05) had a *P* value of 0.06. As shown on exploratory analysis (Figure 2), the most striking reduction in glycated hemoglobin was observed among participants in the diacerein group who had shorter duration of diabetes mellitus. The difference in HbA1c level was −1.3 (95% CI: −2.3 to −0.4) in favor of diacerein (*P* = 0.007) in the <14 years of diabetes duration versus 0.05 (−0.7 to 0.8), with *P* = 0.9, in the 14–30 years in the diabetes duration group. Assessment of HbA1c at baseline and at the end of the trial for each treatment group showed that, in the subgroup with longer duration of diabetes, there was a crude reduction of one percentage point in both the diacerein (9.6 ± 1.7 to 8.7 ± 1.5) and placebo (8.7 ± 1.1 to 7.7 ± 1.3) groups. In the subgroup with <14 years of diabetes duration, diacerein reduced HbA1c by one percentage point (8.7 ± 1.0 to 7.6 ± 1.3), with no appreciable change in the placebo group (8.6 ± 1.1 to 8.9 ± 1.3). Figure 2 shows that both groups of patients, independent of duration of T2DM, had HbA1c levels decreased by about one percentage point at the end of the trial; in the shorter T2DM duration subgroup, the placebo group exhibited a noticeable increase in HbA1c, while in the longer T2DM duration subgroup, there had been a reduction of approximately one percentage point, independently of gender and age. Plotting diabetes duration against HbA1C yielded a Spearman correlation coefficient of 0.42 (*P* = 0.01) for the diacerein group and 0.27 (*P* = 0.12) for the placebo group.

There were no statistically significant differences between groups in fasting glucose, HOMA-IR, and serum lipid levels; renal, hepatic, hematological, and inflammatory parameters; or anthropometric assessment (Table 2).

The results of the inflammatory markers are shown in Table 3. The diacerein group had a 50% increase in the number of participants at the lowest level of TNF-*α* (≤1.46 pg/mL) at the end of the trial, independently of baseline values. Adjusting for confounding factors (the same variables described in Table 2) did not change this result.

Table 4 shows a similar distribution of adverse effects between diacerein and placebo, except for a higher frequency of dark urine in the former. Five other symptoms, including diarrhea and nausea, were more often reported by participants in the diacerein group, but there was no drug-related discontinuation of treatment.

## 4. Discussion

This randomized controlled trial assessed the effect of diacerein (versus placebo) as an adjunctive agent in the treatment of T2DM in patients receiving antidiabetic drugs. Diacerein was able to reduce HbA1c in comparison to placebo (*P* = 0.06), but the difference became statistically significant for participants who had shorter duration of diabetes mellitus. Among participants with long-established T2DM, diacerein and placebo both reduced the HbA1c levels; therefore, there was no effect [0.05 (95% CI: −0.7 to 0.8)]. On the other hand, in participants with shorter duration of diabetes, diacerein was associated with one percentage point reduction, while in the placebo group, there was a noticeable increase in HbA1C level after controlling for age and gender. The effect toward better control of diabetes is consistent with the decrease in TNF-*α* level observed in the diacerein group at the end of the trial. Diacerein reduced the proportion of participants with abnormal levels comparatively to the placebo group, as detected by a 50% increase in the number of participants who migrated to the lowest percentile.

This trial was carried out in T2DM patients with long-established disease and undergoing long-term treatment with metformin, sulfonylureas, or insulin. In two previous studies [9, 10], diacerein was evaluated in small samples, among T2DM patients under the age of 60, who were drug-naïve or receiving metformin as monotherapy for diabetes. Ramos-Zavala et al. [9] evaluated the efficacy of diacerein in 40 drug-naïve patients with newly diagnosed T2DM and HbA1c levels between 7% (53 mmol/mol) and 9% (75 mmol/mol) and found a significant absolute reduction of 1.3% (14.2 mmol/mol) in HbA1c as well as in TNF-*α* and IL-1*β* levels. Villar et al. [10] evaluated 12 patients receiving metformin as monotherapy and found a significant improvement of glycemic control, with reduction of HbA1c and glucose levels. Cardoso et al. [11] selected 84 patients aged less than 75 years, receiving full glucose-lowering treatment, who were randomized to diacerein or placebo with 48 weeks of follow-up. The diacerein group had a 0.35% reduction in HbA1c compared to the placebo group at week 24 of treatment, but this effect was attenuated to a nonsignificant difference at week 48. Participants had a median disease duration of 10 years (interquartile range: 6–18 years). The results of these trials point in the same direction, that is, a reduction in HbA1c in patients who receive diacerein. However, there are striking differences between the present trial and those of Ramos-Zavala and Cardoso, both of which selected patients who were receiving no treatment for diabetes or only monotherapy. Our trial is more similar to that of Cardoso et al. [11]. However, we enrolled patients up to 79 years of age and with longer duration of diabetes and followed participants for a longer time. These differences raised the need to evaluate the effect of diacerein by taking into account the duration of diabetes, which was done in an exploratory analysis.

The reduction in HbA1c levels was not followed by a reduction in HOMA-IR levels, suggesting that the effect of diacerein may have been on insulin secretion. Studies have shown that T2DM is an inflammatory disease. Adipose tissue appears to play a central role in the induction of inflammation, leading to the production of proinflammatory cytokines, particularly TNF-*α* and IL-1*β*. These cytokines induce *β*-cell dysfunction, with subsequent apoptosis and insulin deficiency [4]. However, few controlled studies have tested drugs that play a role in the inhibition of proinflammatory cytokines and their receptors in patients with T2DM. Drugs with anti-inflammatory action may be more effective in the early stages of the disease, when functional impairment of pancreatic *β*-cells is less severe, with a better chance of regaining their function, thus contributing to the main effect of lowering HbA1c levels among those with a shorter duration of diabetes. Malaguti et al. [13], in an experimental study, tested diacerein in nonobese diabetic mice aiming to investigate anti-inflammatory properties and their potential effects on type 1 diabetes. The results showed that diacerein, via modulation of proinflammatory cytokines (IL-1*β* and TNF-*α*), interferes with the onset of type 1 diabetes and may alter the incidence of autoimmune diabetes in treated animals. These findings are consistent with the reduced TNF-*α* observed in this study of T2DM patients. Although autoimmune mediators are not typical of this condition, inflammation may potentially lead to *β*-cell apoptosis. Another experimental study in obese mice suggests that diacerein may be a potential alternative treatment to reverse or improve clinical features of obesity-related T2DM and insulin resistance [14].

The present study has some limitations that should be noted. The size of the sample and the relatively short duration of the intervention, considering the long-standing nature of T2DM, precluded assessment of the long-term efficacy and safety of diacerein. In addition, our findings do not allow us to draw any conclusion about the effects of diacerein among patients with poor metabolic control, since patients with HbA1c ≥ 11.0% (97 mmol/mol) were excluded. Finally, the effect on the subgroup with shorter duration of diabetes should be viewed with caution, as this was a post hoc analysis.

In conclusion, diacerein therapy in patients with long-established T2DM and long-term use of glucose-lowering agents improves metabolic control as measured by HbA1c level and has a favorable impact on the inflammatory profile. Thus, the use of diacerein may be beneficial in counteracting the adverse consequences of diabetes. However, further studies with a larger sample size, including participants exposed to diacerein with shorter and longer duration of diabetes, are needed to confirm these findings.

## Figures and Tables

**Table 1:** Baseline characteristics of participants randomized to diacerein and placebo.

Characteristics	Diacerein (*n* = 35)	Placebo (*n* = 36)	*P* value
Age (years)	62 ± 8.0	59 ± 11.3	0.12
Male	20 (57.1)	27 (75.0)	0.11
Years at school	6.4 ± 4.1	5.6 ± 3.0	0.4
Chronic complications of diabetes	18 (51.4)	13 (36.1)	0.19
Cardiovascular disease	14 (40.0)	13 (36.1)	0.7
Hypertension	29 (82.9)	31 (86.1)	0.7
24 h systolic blood pressure (mmHg)	120.3 ± 11.5	123.1 ± 11.4	0.7
24 h diastolic blood pressure (mmHg)	75.0 ± 8.4	72.3 ± 8.1	0.3
Duration of diabetes (years)	14.0 ± 6.7	14.8 ± 6.6	0.7
Duration of diabetes 14–30 years	19 (54.3)	23 (63.9)	0.4
Use of oral hypoglycemic agents^‡^			
Sulfonylureas	13 (37.1)	12 (33.3)	0.7
Metformin	25 (71.4)	31 (86.1)	0.13
Use of insulin	17 (48.6)	22 (61.1)	0.3
Use of other antidiabetics^#^	1 (2.9)	4 (11.1)	0.17
Metabolic profile			
HbA1c (%)	9.1 ± 1.4	8.6 ± 1.0	0.11
HbA1c (mmol/mol)	76.7 ± 2.4	71.2 ± 2.4	0.11
Fasting glucose (mg/dL)	142.0 ± 69.5	138.7 ± 58.2	0.8
HOMA-IR	5.4 ± 1.0	6.5 ± 1.0	0.4
Lipids			
Total cholesterol (mg/dL)	149 ± 32.7	152.7 ± 34.8	0.6
HDLc (mg/dL)	42.1 ± 7.4	42.8 ± 8.0	0.7
LDLc (mg/dL)	73.0 ± 35.6	78.5 ± 35.5	0.3
Triglycerides (mg/dL)	200.5 ± 124.4	202.4 ± 210.5	1.0
Renal			
Creatinine (mg/dL)	0.93 ± 0.15	0.87 ± 0.17	0.11
eGFR (mL/min/1.73 m^2^)	77.0 ± 13.1	83.5 ± 23.5	0.13
Urinary albumin/creatinine ratio (mg/24 h)	9.9 ± 6.6	10.8 ± 6.8	0.6
Hepatic			
ALT (units/L)	23.5 ± 11.1	23.1 ± 11.3	0.9
AST (units/L)	24.7 ± 11.8	21.9 ± 8.1	0.2
Gamma-glutamyltransferase (units/L)	43.4 ± 26.4	36.1 ± 20.6	0.2
Hematologic/inflammatory			
Hematocrit (%)	42.0 ± 4.9	41.7 ± 4.3	0.8
Platelet count (×10^9^ cells/L)	215.0 ± 55.7	221.8 ± 60.0	0.6
C-reactive protein (mg/L)	7.0 ± 6.2	9.0 ± 16.4	0.5
Anthropometric assessment			
Weight (kg)	80.3 ± 19.8	78.7 ± 13.1	0.7
Height (m)	1.62 ± 0.10	1.59 ± 0.07	0.16
Body mass index (kg/m^2^)	30.8 ± 6.9	31.3 ± 5.2	0.7

Data expressed as *mean* ± *SD* and *n* (%). ^‡^Numbers exceed 100% due to use of more than one agent. ^#^Alpha-glucosidase inhibitors, meglitinides, DPP4 inhibitors, and amylin mimetics.

**Table 2:** Mean values during the 12-week treatment and adjusted differences in mean changes between diacerein and placebo for the trial outcomes (mean ± SE).

	Diacerein (*n* = 35)		Placebo (*n* = 36)		Adjusted difference in mean change (diacerein-placebo)^∗^	*P* value
	End of trial	Change from baseline	End of trial	Change from baseline		
Metabolic profile						
HbA1c (%)	8.1 ± 0.2	−0.98 ± 0.2	8.1 ± 0.2	−0.52 ± 0.2	−0.98 (−2.02 to 0.05)	0.06
HbA1c (mmol/mol)	65.9 ± 2.6	−10.8 ± 2.4	65.5 ± 2.6	−5.7 ± 2.3	−10.8 (−22.0 to 0.5)	0.06
Fasting glucose (mg/dL)	146.3 ± 13.1	4.3 ± 11.9	150.4 ± 12.9	11.8 ± 11.8	−19.8 (−78.3 to 38.5)	0.5
HOMA-IR	5.3 ± 0.8	−0.18 ± 0.7	6.2 ± 0.8	−0.27 ± 0.7	1.0 (−2.6 to 4.6)	0.6
Lipids						
Total cholesterol (mg/dL)	148.7 ± 5.5	−0.3 ± 4.6	151.1 ± 5.2	−1.6 ± 4.5	−0.12 (−22.9 to 22.6)	1.0
HDLc (mg/dL)	41.3 ± 1.3	−0.8 ± 0.9	42.8 ± 6.9	−0.03 ± 0.8	−2.2 (−6.4 to 2.1)	0.3
LDLc (mg/dL)	79.9 ± 5.1	6.8 ± 6.1	78.2 ± 5.0	−3.2 ± 6.0	20.1 (−10.4 to 50.5)	0.19
Triglycerides (mg/dL)	153.1 ± 17.7	−47.4 ± 23.2	164.7 ± 17.4	−37.7 ± 22.9	−78.7 (−193.4 to 36.1)	0.18
Renal						
Creatinine (mg/dL)	1.0 ± 0.04	0.06 ± 0.03	1.0 ± 0.04	0.03 ± 0.03	0.12 (−0.03 to 0.26)	0.11
eGFR (mL/min/1.73 m^2^)	73.9 ± 3.2	−3.1 ± 1.9	81.7 ± 3.2	−1.8 ± 1.9	−7.7 (−17.3 to 1.9)	0.12
Urinary albumin/creatinine ratio (mg/24 h)	24.0 ± 5.6	14.1 ± 5.2	27.3 ± 5.5	16.5 ± 5.2	6.0 (−19.4 to 31.4)	0.6
Hepatic						
ALT (units/L)	24.4 ± 2.4	0.9 ± 1.6	25.1 ± 2.4	2.0 ± 1.5	0.5 (−6.5 to 7.5)	0.8
AST (units/L)	27.1 ± 2.3	2.4 ± 1.5	26.2 ± 2.3	4.3 ± 1.5	−0.6 (−8.0 to 6.8)	0.9
Gamma-glutamyltransferase (units/L)	44.9 ± 4.9	1.5 ± 2.4	39.3 ± 4.8	3.2 ± 2.3	−7.7 (−19.3 to 4.0)	0.19
Hematologic/inflammatory						
Hematocrit (%)	42.7 ± 0.8	0.8 ± 0.8	43.4 ± 0.8	1.7 ± 0.8	0.5 (−3.6 to 4.5)	0.8
Platelet count (×10^9^ cells/L)	230 ± 10.2	15.03 ± 7.3	236 ± 10.0	14.6 ± 7.2	−5.1 (−41.6 to 31.4)	0.8
C-reactive protein (mg/L)	9.0 ± 2.5	2.0 ± 3.3	12.3 ± 2.5	3.3 ± 3.2	−9.8 (−25.3 to 5.7)	0.2
Anthropometric assessment						
Weight (kg)	77.6 ± 2.6	−2.7 ± 0.7	77.3 ± 2.6	−1.4 ± 0.7	−2.4 (−5.6 to 0.9)	0.16
Body mass index (kg/m^2^)	29.8 ± 1.0	−1.0 ± 0.2	30.8 ± 1.0	−0.6 ± 0.2	−0.8 (−2.0 to 0.4)	0.19

^∗^Mean adjusted for age, gender, duration of diabetes, and addition of new antidiabetic drugs.

**Table 3:** Effect of diacerein on inflammatory markers at end of trial.

	Diacerein (*n* = 35)	Placebo (*n* = 36)	RR (95% CI)^∗^	*P* value
*α* − Tumor necrosis factor ≤ 1.46 (pg/mL)	23 (65.7)	14 (38.9)	1 (1.04–2.1)	0.03
Interleukin − 1*β* > 1.23	8 (22.9)	15 (41.7)	0.7 (0.4–1.3)	0.3
Interleukin − 6 ≤ 10.7	17 (48.6)	14 (38.9)	1.4 (0.9–2.2)	0.19
Interleukin − 8 ≤ 29.53	6 (17.1)	2 (5.6)	3.1 (0.7–14.8)	0.15
Interleukin − 10 ≤ 5.25	10 (28.6)	5 (13.9)	1.8 (0.7–4.6)	0.19
Leptin ≤ 10.3	11 (31.4)	6 (16.7)	1.4 (0.8–2.6)	0.3
Selectin ≤ 53.4	6 (17.1)	16 (44.4)	0.6 (0.3–1.2)	0.14
Adiponectin ≤ 11.8	15 (42.9)	17 (47.2)	0.8 (0.5–1.2)	0.4

Data expressed as *n* (%) and relative risk (95%CI). ^∗^Relative risk adjusted for baseline values of the inflammatory markers.

**Table 4:** Frequency of adverse events in each treatment group.

Adverse events	Diacerein (*n* = 35)	Placebo (*n* = 36)	*P*
Nausea	6 (17.1)	3 (8.3)	0.3
Vomiting	1 (2.9)	4 (11.1)	0.2
Diarrhea	10 (28.6)	6 (16.7)	0.2
Abdominal pain	4 (11.4)	4 (11.1)	1.0
Dark urine	21 (7.4)	8 (2.9)	0.001
Others	5 (14.3)	0	0.03

Data expressed as *n* (%).
